# Surface Modification Techniques of Titanium and its Alloys to Functionally Optimize Their Biomedical Properties: Thematic Review

**DOI:** 10.3389/fbioe.2020.603072

**Published:** 2020-11-11

**Authors:** Tong Xue, Shokouh Attarilar, Shifeng Liu, Jia Liu, Xi Song, Lanjie Li, Beibei Zhao, Yujin Tang

**Affiliations:** ^1^School of Metallurgical Engineering, Xi’an University of Architecture and Technology, Xi’an, China; ^2^Department of Pediatric Orthopaedics, Xin Hua Hospital Affiliated to Shanghai Jiao Tong University School of Medicine, Shanghai, China; ^3^State Key Laboratory of Metal Matrix Composites, School of Material Science and Engineering, Shanghai Jiao Tong University, Shanghai, China; ^4^Affiliated Hospital of Youjiang Medical University for Nationalities, Baise, China; ^5^Chengsteel Group Co., Ltd., HBIS Group Co., Ltd., Chengde, China

**Keywords:** titanium, surface modification, implant materials, osteogenesis properties, antibacterial function

## Abstract

Depending on the requirements of specific applications, implanted materials including metals, ceramics, and polymers have been used in various disciplines of medicine. Titanium and its alloys as implant materials play a critical role in the orthopedic and dental procedures. However, they still require the utilization of surface modification technologies to not only achieve the robust osteointegration but also to increase the antibacterial properties, which can avoid the implant-related infections. This article aims to provide a summary of the latest advances in surface modification techniques, of titanium and its alloys, specifically in biomedical applications. These surface techniques include plasma spray, physical vapor deposition, sol-gel, micro-arc oxidation, etc. Moreover, the microstructure evolution is comprehensively discussed, which is followed by enhanced mechanical properties, osseointegration, antibacterial properties, and clinical outcomes. Future researches should focus on the combination of multiple methods or improving the structure and composition of the composite coating to further enhance the coating performance.

## Introduction

With the growing maturity of medical technologies, it is found that implanting biomaterials into the human body is an excellent way to treat some of the orthopedic and dental diseases (Lausmaa et al., 1990; Ohthuki et al., 1999). The commonly used metallic biomaterials are titanium (Ti) and its alloys (Wang et al., 2009; Guo et al., 2013; Jemat et al., 2015; Hafeez et al., 2019), 316L stainless steel (Singh et al., 2018), and cobalt-based alloys (Wang et al., 2014). Apart from these, shape memory alloys like magnesium (Mg) (Kirkland et al., 2010), NiTi (Bansiddhi et al., 2008; Wang et al., 2016, Wang et al., 2018; Liu et al., 2020a, b), and tantalum (Ta) are also potential candidates in biomedical applications (Balla et al., 2010). For the first time Ti was discovered in the 1790s (Chouirfa et al., 2019). Nowadays, due to its high specific strength, strong corrosion resistance, and excellent biocompatibility (Jemat et al., 2015; Niinomi et al., 2016; Shi et al., 2017; Rabadia et al., 2018, 2019; Ran et al., 2018; Hafeez et al., 2020; Wang L. et al., 2020), titanium and its alloys have been widely used in the biomedical field (Wang et al., 2017), among which Ti-6Al-4V alloy applications account for more than 50% (Hu et al., 2012; Ding et al., 2016; Zhang et al., 2017). Although their beneficial properties (Matter and Burch, 1990), titanium and its alloys are considered as inert metals and cannot properly stimulate the proliferation of osteoblasts and bone cells (Zhu et al., 2016; Xiao et al., 2017; Souza et al., 2019). In addition, most of the failures are caused by implant-related infections, thus, lots of researches have been focused on improving the antibacterial ability of titanium implants (Yousefi et al., 2017; Ding et al., 2019; Liu et al., 2019; Wang et al., 2020). The exposed titanium alloy cannot resist the wear triggered by the relative movement between the implant and the bone, and external force and body fluid immersion will cause the disappearance of the passive film on the titanium alloy surface, resulting in a decrease in its corrosion performance (Zhang and Chen, 2019). The above problems can be solved by improving the surface properties of titanium and its alloys. Therefore, various surface modification methods have been used to improve the biological function, wear, and corrosion resistance of implants. In the last decade, coatings have been using in multiple applications to modify the surface of implants and, in some cases, to create new surfaces with exceptional properties that are very different from uncoated materials (Zhong, 1999, 2001; Wang et al., 2015; Wang et al., 2017; Gu et al., 2019). Furthermore, many studies proved that surface modifications techniques can minimize the bacterial adhesion on the implant substrate. They can also inhibit the biofilm formation and provide the effective bacterial removal, thus improving the performance of implanted biomaterials (Asri et al., 2017; Awad et al., 2017; Ahn et al., 2018; Zhang et al., 2020).

**FIGURE 1:**
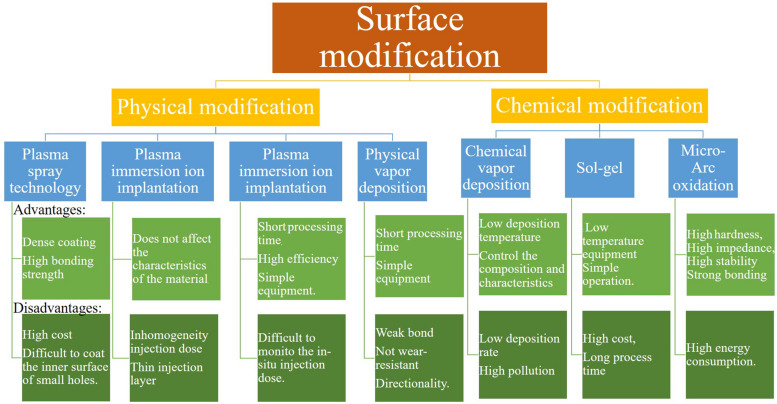
Surface treatment methods with their advantages, disadvantages, and applications (Vahabzadeh et al., 2015; Wang et al., 2015; Asri et al., 2017; Azari et al., 2019; Chouirfa et al., 2019; Kaur and Singh, 2019; Souza et al., 2019; Thangavel et al., 2019; Youn et al., 2019; Xia et al., 2020).

This review thematically focuses on surface modifications technologies such as plasma spray, plasma immersion ion implantation (PIII), plasma immersion ion implantation and deposition (PIII&D), physical vapor deposition (PVD), chemical vapor deposition (CVD), sol-gel, and micro-arc oxidation (MAO) methods. These methods are divided into two major parts: physical modification and chemical modification techniques. In the chemical methods, the surface is dipped into chemically active solutions, while in physical methods the surface is exposed to highly energetic charges or other physical species like a flame, plasma, etc. Certain technologies can have the involvement of multiple physical and chemical processes. Thus, it is impossible to strictly separate physical and chemical methods. The classification mainly depends on the main idea behind each technology. Moreover, this article summarizes the osteogenic and antibacterial properties achieved through surface technologies on Ti-based implant materials from these two aspects and provides a comprehensive incite to improve the surface techniques to manufacture the modern implant materials with improved properties. Figure 1 shows all the surface treatment methods along with their pros and cons.

## Physical Modification

The main idea of the physical modification method in Ti-based alloys is to treat and change the surface ultrastructure, and these methods include plasma spray technology, PIII, PIII&D, and PVD. The physical modification method is relatively cheap, and the preparation method and mechanism are simple. Correspondingly, the bonding force of the coating is weak, and it is slightly insufficient in the preparation of complex samples. Table 1 gives a comparison of the main results of different physical methods.

**TABLE 1:** Main results of physical methods application on titanium and its alloys.

Methods	Coating quality	Process rate	Bond strength	Osteogenesis and antibacterial function	Applications
Plasma spray technology (Hanawa, 2019; Ke et al., 2019)	Dense coating, high bonding strength, difficult to oxidize spray material	Not available	20–80 MPa	Enhanced osseointegration, osteoblast proliferation and rapid bone repair.	Wide range of materials, suitable for a variety of coatings
Plasma immersion ion implantation (Yu et al., 2017; Shanaghi and Chu, 2019b)	Inert to surface thin injection layer, good biocompatibility.	Not available	Not available	Inhibition of *Staphylococcus aureus* and *Escherichia coli (E. coli)*	Used for large, heavy. and complex shaped workpieces
Plasma immersion ion implantation and deposition (Yang et al., 2007; Yu et al., 2017)	Easy composition control, improved density and adhesion, suitable for three-dimensional and complex surfaces	≈30–40 nm min^–1^ in thickness	Very high	Rapid osseointegration and continuous biomechanical stability, reduction of gram-negative *E. coli* and *Pseudomonas aeruginosa*	Used for precision parts with high added value to improve the wear resistance
Physical vapor deposition (Behera et al., 2020; Liu et al., 2020)	Uniform and dense film, strong bonding force	≈25–1,000 nm min^–1^ in thickness	Moderate	Surface modification to increase the contact area, good blood compatibility	Suitable for preparation of special functional composite membrane

### Plasma Spray Technology

**FIGURE 2:**
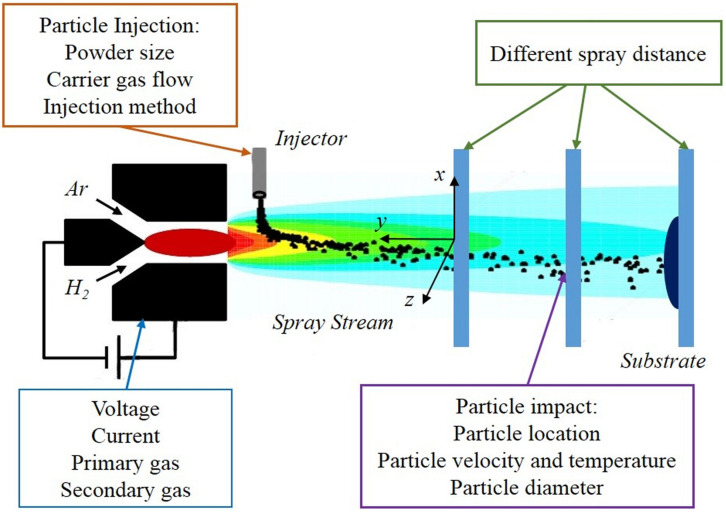
Related parameters and important variables of plasma spraying. Reproduced from Zhu et al. (2020) with permission.

Plasma spray technology is a thermal spraying technique using plasma arc as the heat source, and it has been widely used to form coatings with excellent physical, chemical, and mechanical properties (Karthikeyan et al., 1997; Shaw et al., 2000), especially in the biomedical field. As shown in Figure 2, many parameters involved in this method, which can potentially affect the microstructure and properties of coatings, among them porosity is the most significant factor which determines the coating quality.

**FIGURE 3:**
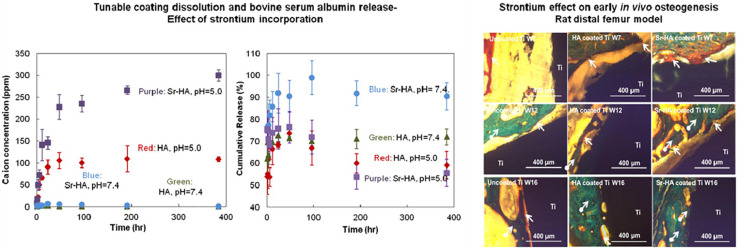
The evaluation of stability and new bone formation of plasma sprayed HA coating. Reproduced from Vahabzadeh et al. (2015) with permission.

Hydroxyapatite (HA) coating is used to improve osteoconductivity and enhances osseointegration. Kotian et al. (2017) analyzed the production of HA coatings on Ti and Ti-6Al-4V under different plasma atmospheres. They proved that the atmosphere has a substantial influence on the composition, crystallinity, and the formation of microcracks of HA-coated implants. In order to obtain high-quality coatings, researchers need to control the temperature of the plasma gas to reduce microcracks. Besides, the atmosphere with argon and nitrogen gases showed the highest degree of crystallinity. Furthermore, according to Liu Y.-C. et al. (2020), a new vapor-induced pore-forming atmospheric plasma spraying (VIPF-APS) technique has great potential for producing bioactive porous HA coating which enhances osteoblast attachment and differentiation. Apart from the plasma spray technology, other strategies have been considered to improve the overall performance of the coating. Meanwhile, a new double-layer HA/Al_2_O_3_-SiO_2_ coating was put forward by Ebrahimi et al. (2018), compared to monolayer HA, it has improved cell behavior and biocompatibility. Vahabzadeh et al. (2015) and Cao et al. (2019) doped Sr (Mg and Sr) into HA coating. In Figure 3, the formation of steroids is evident in the Sr-HA coating, which indicates that the bone regeneration of the Sr-HA coating is accelerated compared to uncoated Ti and HA coating implants. As for (Mg, Sr)-HA, on the fifth day, the visible cell adhesion prove its good biocompatibility on the surface of the coating, and it also showed high bonding strength. In another study, MgO, Ag_2_O, and gradient HA were mixed in order to improve the biological and antibacterial properties (Ke et al., 2019). This novel method improves osseointegration and decreases the possibility of failure due to loosening or infection. Besides, Otsuka et al. (2016) clarified that due to the acceleration of dissolution at the interface, the delamination life of the HA coating immersed in the simulated body fluid (SBF) is shortened. Therefore, the delamination behavior of extracorporeal circulation should be considered to extend the service life of HA coatings.

Researchers investigated the composite coatings for a decade, trying to improve the tribological behavior of implants, Ganapathy et al. (2015) prepared Al_2_O_3_ −40 wt.%8 YSZ on the biomedical grade Ti-6Al-4V alloy used for total joint prosthetic components through plasma spray. Another method, combined with the mutual effect of ceramics and metallic materials, have been investigated by Veerachamy et al. (2018). According to their research, Al_2_O_3_ +13 wt.% TiO_2_/-YSZ BL can be deemed as a suitable coating on Ti-6Al–4V because of its high antibacterial activity and superior cell compatibility. Furthermore, the bioactive glass-ceramic coating named M2 coating (including CaO–MgO–SiO_2_) on Ti-6Al-4V alloy has shown good performance *in vitro*. In order to figure out its performance in osteogenesis and osseointegration, Zhang et al. (2019) implanted it into rabbits, it was verified that the M2 coated Ti-6Al-4V was provided with the better biological performance *in vivo* and could probable replace HA coating to repair load bearing bone implants. Many new coating materials have received extensive attention. For instance, tricalcium magnesium silicate is recommended as a new coating, which has almost the same thermal expansion properties as Ti-6Al-4V, also it has the potential to enhance the corrosion and biological behavior of permanent metallic implants (Maleki-Ghaleh et al., 2015). At the same time, other metal elements with excellent bio-properties like tantalum (Kuo et al., 2019), have been deposited on titanium alloy implants.

Plasma spray technology provides a cost-effective, straightforward, and reliable approach to prepare coatings on titanium alloys. The gas atmosphere and temperature of plasma spraying will affect the thermal stress and crystallinity of the coating, which will affect the osteogenic activity and other properties. On the one hand, as the conventional coating material, HA needs to be upgraded by improving the production process or with doping new elements. On the other hand, novel coatings like metal composites should be considered. Although it was initially found that plasma sprayed TiO_2_ and ZrO_2_ coatings have good biological activity and biocompatibility, the related mechanisms still need to be further explored. In addition, the plasma spraying temperature is extremely high, and the coating encounters large thermal stresses. Special attention should be paid to the bonding force between the coating and the substrate. Also, it still requires some improvement in the preparation of coatings on small and special-shaped workpieces.

### Plasma Immersion Ion Implantation

Since the PIII technique enables to embed a great variety of elements into the near-surface region of the various substrates, it offers unique advantages for surface modification technologies of biomaterials (Lin et al., 2019). The most valuable feature of PIII is that the concentration and depth distribution of the implanted ions in the substrate can be strictly controlled by adjusting the implantation parameters (Jin et al., 2014). In addition, it has been demonstrated that it can enhance the hardness, corrosion resistance, wear resistance, bioactivity, and antibacterial properties of biomaterials (Chen et al., 2020).

**FIGURE 4:**
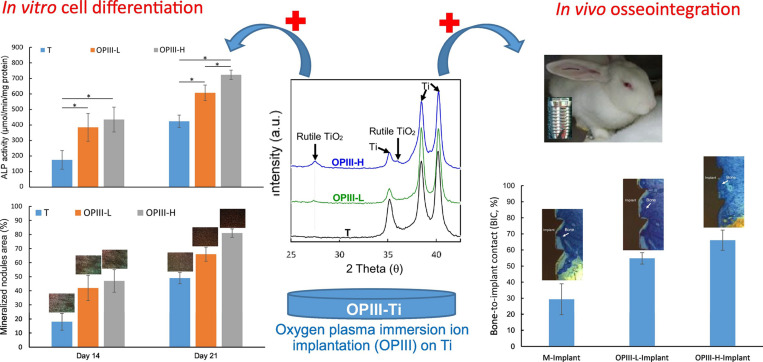
The illustration of the presence of rutile phase TiO_2_, which enhances the osteocytic differentiation and osseointegration of dental Ti implants *in vivo*. Reproduced from Chen et al. (2020) with permission.

As the most common surface coating in Ti-based alloys, TiO_2_ has attracted attention in the PIII method. PIII method and optical emission spectrometry (OES) was used to produce TiO_2_, which has the potential to improve the osseointegration of implants due to its super hydrophilicity (Lin et al., 2019). Shiau et al. (2019) and Chen et al. (2020) investigated the parameters of O-PIII respectively, the former proved that the applied voltage during O-PIII treatment promote blood-clotting and platelet activation, as shown in Figure 4, the latter indicates that the use of higher doses of oxygen ions can improve osteocytic differentiation and osseointegration of dental Ti implants *in vivo*. In addition to O-PIII, nitrogen plasma immersion ion implantation (N-PIII), carbon plasma immersion ion implantation (C-PIII), etc. were also widely used in fabricating coatings. Nitrogen was incorporated into TiO_2_ coatings by N-PIII, which could effectively reduce the viability of bacteria in visible light (Zheng et al., 2020). Different from N-PIII, C-PIII was used to preparing coatings with increased mechanical properties and corrosion resistance (Shanaghi and Chu, 2019a). Unfortunately, it could also release the Ni element of NiTi alloys in the SBF solution (Shanaghi and Chu, 2019b).

**FIGURE 5:**
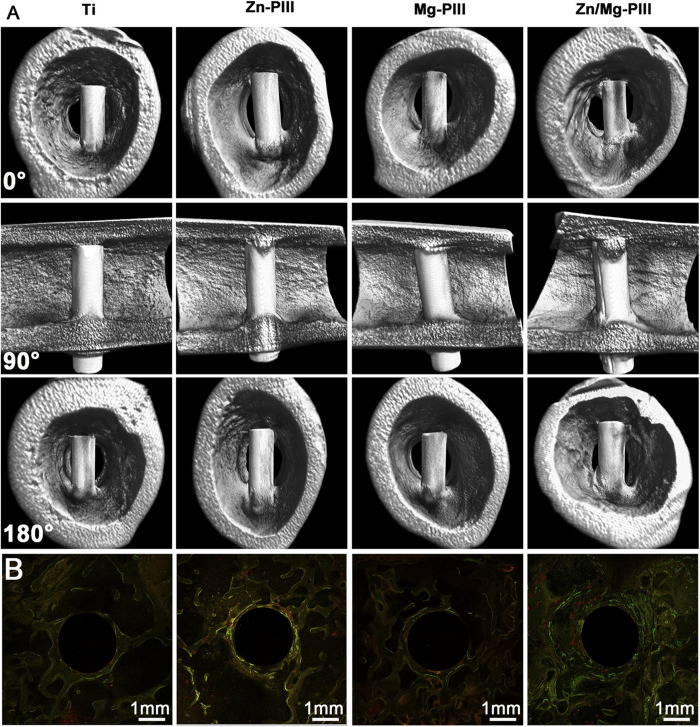
Twelve weeks after implantation, newly formed around Zn/Mg-PIII implants and its sequential fluorescent labeling images. Reproduced from Yu et al. (2017) with permission. **(A)** Micro-CT 3D images of new bone formation around various implants in rabbit femur. **(B)** Sequential fluorescent labeling images of newly-formed bone around various implants in rabbit femoral condylar: Alizarin red S (red), tetracycline (yellow), Calcein (green).

Besides, the TiN thin film can be formed on Ti-6Al-4V by N-PIII method (Huang et al., 2019), which could positively affect the surface hardness, corrosion resistance, cell responses, and antibacterial adhesion. Furthermore, Xu et al. (2015) added Ag into TiN films as an antibacterial agent, which has good cytocompatibility and retains the required mechanical properties. The Zn-implanted Ti exhibits excellent osteogenic activity and partly antibacterial effect. It is worth noting that the depth profile of zinc in CP-Ti resembles a Gaussian distribution (Jin et al., 2014). Interestingly, Yu et al. (2017) developed dual Zn/Mg ion co-implanted titanium (Zn/Mg-PIII). Zinc is considered as an important and necessary trace element for bone metabolism and production, also Mg plays a critical role in the adhesion of osteoblasts and osteoblasts to orthopedic implants. Thus, due to the beneficial combination of Zn/Mg, the Zn/Mg-PIII implants present good osteoinductivity, pro-angiogenic and antibacterial effects and as shown in Figure 5, these implants can increase the rate of osseointegration and maintain biomechanical fixation.

In summary, with the ability to control the concentration and depth distribution of implanted ions, PIII shows the potential to implant single or multiple metal ions according to demand. Cell differentiation and osseointegration can be enhanced by injecting designated oxygen, nitrogen, or carbon ions. Additionally, O-PIII, N-PIII, C-PIII, etc. could contribute to essential elements for biocompatibility. Therefore, future research should focus on the procedures to achieve reasonable implantation of multiple metal ions by adjusting the PIII process parameters and to reduce the cytotoxicity caused by metal ion release.

### Plasma Immersion Ion Implantation and Deposition

**FIGURE 6:**
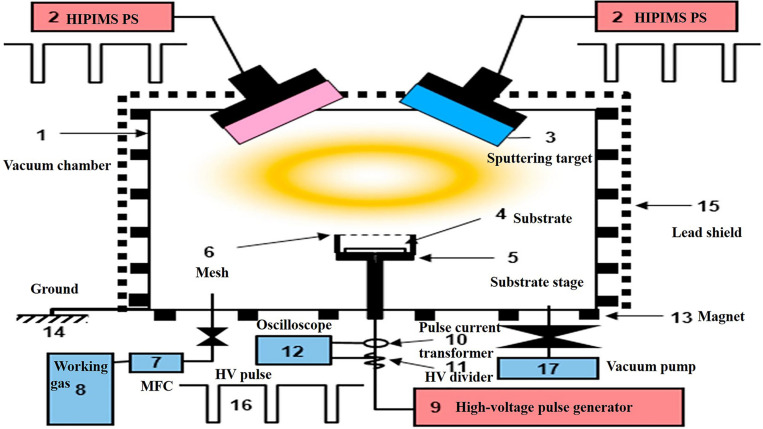
A schematic representation of the PIII&D instrument. Reproduced from Hwang et al. (2019) with permission.

The PIII&D method, invented in 1987 by Conrad et al. (1987), it has become a routine surface modification method. It has the advantage to levitate the retained dose levels that were limited by the sputtering because of ion implantation. Therefore, using PIII&D with relatively low cost, a three-dimensional film with strong adhesion, thick and without stress is possible to be produced (Yang et al., 2007). The schematic process of PIII&D was shown in Figure 6.

**FIGURE 7:**
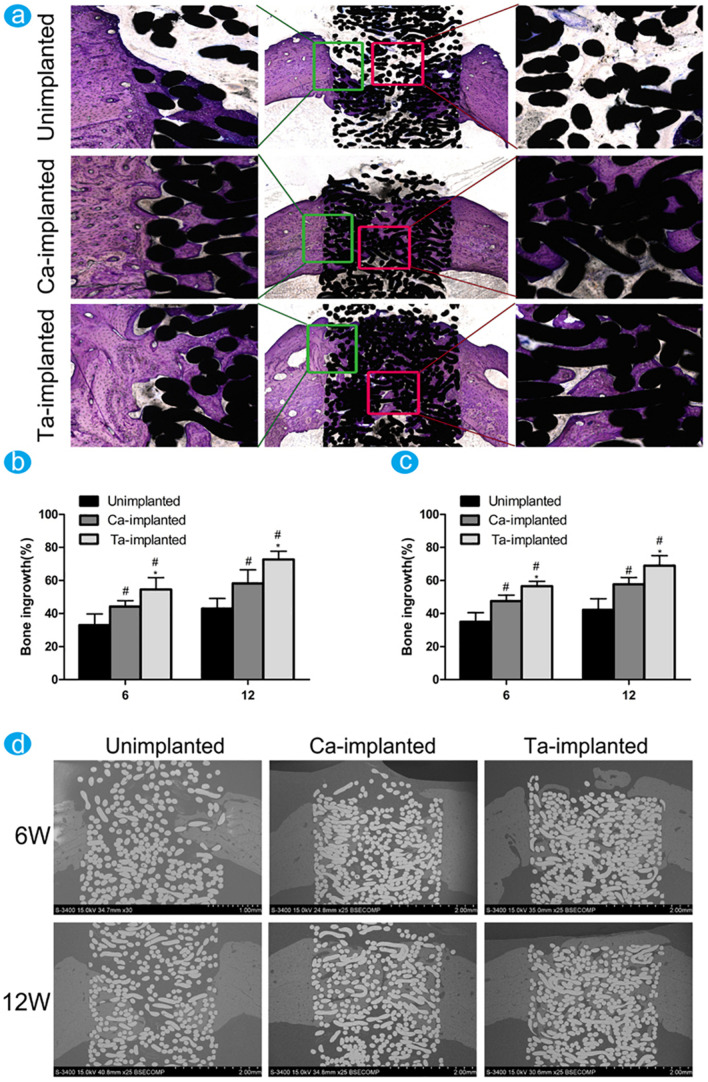
The difference of new bone ingrowth was evaluated through histological observation and histomorphological measurement. Reproduced from Wang et al. (2016) with permission. **(a)** Undecalcified sections of samples were stained with toluidine blue at 12 weeks. The percentage of new bone ingrowth and pores in different EPT implants measured from toluidine blue staining **(b)** and back scattered SEM images **(c)** at 6 and 12 weeks. **(d)** The back scattered SEM images of new bone around and inside pores of EPT implants at 6 and 12 weeks.

Facing a crucial problem namely thrombosis, blood-contacting biomaterials need to form an interface between the material and blood. Yang et al. (2007) modified the surface characteristics of biomaterial with the functional inorganic films of Ti–O, a-C: N:H, and Si–N synthesized using PIII-D, which can prevent the platelet adhesion/activation. Later in 2013, Ca film deposition has been completed on Ti for the applications of osseointegration in artificial components (Ueda et al., 2013), which is resulted in the formation of a well adherent Ca film. PIII&D was also used to improve the cell response to titanium, Mg–Ag PIII&D treated Ti not only can inhibit the adhesion and proliferation of *Escherichia coli* bacteria but also promote the initial adhesion and alkaline phosphatase (ALP) expression of MG63 cells (Cao et al., 2014). Concurrently, an excellent compromise between the biocompatibility and cytotoxicity of incorporated metals (like Cu, Mn, etc.) is still required. Copper, a trace element that also exists in the human tissues, has a well-known antimicrobial activity. Hempel et al. (2014) testified that Cu doped and coated Ti can prevent and treat implant-associated infections. It’s worth noting that the surface of overdosed Cu-bearing Ti exhibits negative biocompatibilities (Yu et al., 2016), except for the Cu coating. Yu et al. (2017) investigated a stable Mn ion release on Ti, showing significantly enhanced osteogenesis-related gene expressions and providing a better understanding of relationships between the doped element and biological properties which are caused by additive induction. Aim to solve the bio-inertness of Ti, Ta-implanted entangled porous titanium (EPT) was constructed by the PIII&D method (Wang et al., 2016). As shown in Figure 7, compared with Ca-implanted EPTs, Ta-implanted EPTs shows more stable and continuous effects in the long-term utilization. Another study has deposited zirconium oxide nanostructured coating on the Ti-6Al-4V surface to improve the tribological properties (Saleem et al., 2017). Apart from these coatings, the penetration of nitrogen ions can also be used to support the stability of phospholipid artificial membranes (SLBs) with enhanced biocompatibility (Cisternas et al., 2020). Targeting improvement on the corrosion resistance and prolong the lifespan of Ti, carbon film deposition was accomplished by using a PIII&D system. Santos et al. (2019) verified the desirable properties of carbon films as coating, it can protect the titanium alloy tubes and also can provide new ideas in biology.

In summary, PIII&D technologies are widely used to form metal coatings on titanium and its alloys. Injecting metal ions into the surface of the Ti substrate through the PIII&D technology, since the metal phase tends to act as an anode to release metal ions, the antibacterial properties of the material can be improved. The PIII&D method generally deposits a single metal element on the titanium substrate, but the balance between toxicity and biocompatibility must be considered. PIII&D technology overcomes the apparent linearity problem of other physical deposition methods, and it is suitable for surface modification of complex-shaped workpieces but its biological safety should also be scrutinized. In the future, the deposition of multiple metal elements or carbon nanomaterials must be investigated to further enhance the biocompatibility of the coatings.

### Physical Vapor Deposition

Physical vapor deposition implies a physical coating strategy, involving the evaporation of solid metal under the vacuum environment and depositing it on a conductive substrate (Hauschild et al., 2015). Generally, vacuum evaporation, ion plating, and sputter coating, etc. are among the main methods of PVD. Among them, magnetron sputtering technology has been extensively studied and it results in the formation of high-quality films over a large area and at a relatively low substrate temperature (Nemati et al., 2018; Hamdi et al., 2019).

In the biomedical field, TiN coating concurrent with its favorable biocompatibility can be used as a desirable blood-contacting material. Prachar et al. (2015) compared the properties of TiN with ZrN on pureTi, Ti-6Al-4V, and Ti35Nb6Ta titanium alloys. It was confirmed that TiN has higher cell colonization than ZrN. Furthermore, their color solves the issue of aesthetics in oral implantology because the color of these coatings prevents Ti visibility through the gingiva. Hussein et al. (2020) deposited TiN on Ti20Nb13Zr via cathodic arc PVD. The coated alloys show better corrosion protection behavior in both SBF and the artificial saliva medium. Wu et al. (2019) used the high-power impulse magnetron sputtering (HiPIMS) method which has high peak current and maximum power to deposit TiN on TiAl6V4. The 110 A deposited coating exhibits the highest cell viability. However, the biocompatibility of surface-modified Ti alloys mainly depends on the nitrogen content of the film, therefore in the work of Nemati et al. (2018), TixNy are applied to Ti-6Al-4V substrates as thin films. They controlled the nitrogen partial pressure and prepared samples under a mixed atmosphere of Ar and N_2_. Greater mechanical properties, corrosion resistance, and biocompatibility occurred with the upgrade of the N/Ti ratio. In the work of Bahi et al. (2020), two types of coatings were studied: TiN as the top layer, while the upper layer of the others was TiO_2_ with two different oxygen content. The TiN presents the best tribological performance in the multilayered film condition when its surface slide against the bovine cortical bone. Some researchers (Cui et al., 2019) found that compared to TiN and ZrN coatings, partial replacement of Ti atoms with Zr provides excellent wear resistance and fracture toughness. The TiZrN graded coating which was prepared by Cui et al. (2019) is suitable for artificial joint applications that can endure large loads and resist serious wear conditions. Besides, Hauschild et al. (2015) put the Ag-coated cementless stem into the canine model and showed osseous integration *in vivo*, where toxic side effects did not appear. Afterward, Ag-doped NiTi (NiTi/Ag) coatings were prepared on pure Ti substrates by Thangavel et al. (2019). The NiTi/Ag coating with 3 at. % Ag showed the highest cell viability of human dermal fibroblast neonatal cells and showed a well-grown actin filament network. YSZ was deposited onto the titanium substrate in the study of Kaliaraj et al. (2016) unfortunately this coating couldn’t inhibit bacterial growth but it could enhance the blood protein adhesion. Tantalum pentoxide nanotubes (Ta_2_O_5_ NTs) were prepared on biomedical grade Ti-6Al-4V alloy by Sarraf et al. (2017), results of the SBF tests exhibited that on the first day of immersion, a bone-like apatite layer has already formed on the coating of the nanotubular array, indicating the importance of nanotubular configuration for *in vitro* biological activity.

Nowadays, multi-layered coatings with prominent osseointegration properties and mechanical strength have become focused research areas. In this respect, HA bioceramics have good biocompatibility but the mechanical strength is weak. Hence, Hamdi et al. (2019) prepared triple-layered HA/Al_2_O_3_/TiO_2_ coating on Ti-6Al-4V alloys. In this work, HA plays a critical role in biocompatibility, while others improve the corrosion behavior of the substrate, which prevents the entry of active ions from body fluids onto the surface. Chen et al. (2019) deposit a novel bio-functional bilayer coating consist of calcium phosphate and magnesium (CaP-Mg) on Ti. The CaP coating could inhibit the releasement of Mg, while the alkaline environment caused by Mg degradation has the potential to reduce the bacterial viability. Furthermore, BCP as a kind of CaP is the mixture of β-TCP, Behera et al. (2020) proved that BCP-TiO_2_ film can be beneficial to improve the biological performance of implants. Nowadays, the development of amorphous carbon (a-C) films has received a lot of attention, Liu et al. (2020) successfully deposited Zr/a-C gradient multilayer films (GMFs), it comprised of three distinct layers. Zr/a-C GMFs modified Ti shows upgraded wettability, enhancing the proliferation and adhesion of osteoblast cells.

To sum up, PVD is used as a mature method to form a nearly perfect adhering layer of materials, which does not disintegrate nor affects the surface topography and shows good tribological properties. Due to the mismatch of the coefficient of thermal expansion between the coating and the substrate, their bonding force is weak, which limits the applications of this type of coating. At present, the transition layer or gradient coating deposition methods are generally used in order to reduce the mismatch of the crystal lattice and thermal stress between the coating and the substrate, thereby enhancing its binding force. TiN coatings with different element doped compositions seem to be the further research direction at present. It is necessary to consider the cell cytotoxicity, adhesion, activity, and antibacterial properties of the newly designed coating composition. In addition, multi-layered coatings could provide proper performance, while researchers should rationally design multi-layer structures to maximize their respective advantages and avoid the possible adverse effects.

## Chemical Modification

The chemical modification changes the chemical properties of the carrier surface to produce specific interactions between cell surface molecules, which not only affect the cell surface properties but also cause closely related changes in the internal structure and function of cells. Chemical modifiers are relatively complex in preparation mechanisms and they are expensive. Current research focuses on composition control, multilayer structure design, multi-scale coatings, or coatings with novel surface morphologies. Table 2 gives a comparison of the main findings of different chemical methods.

**TABLE 2:** Main results of chemical methods application on titanium and its alloys.

Methods	Coating quality	Process rate	Bond strength	Osteogenesis and antibacterial function	Applications
Chemical vapor deposition (Du et al., 2016; Youn et al., 2019)	Good controlling on composition and characteristics of the film, flexibility	Deposition by chemical reaction at a high temperature	Not available	It positively affects the proliferation and activity of osteoblast-like cells, sterilization efficiency against *E. coli*	Used for complex workpieces and inner hole coating
Sol-gel (El hadad et al., 2020; Ziabka et al., 2020)	Easy to prepare uniform multi-component oxide film and quantitative doping, effective control on composition and microstructure	Several steps for the preparation of sol-gel, transfer of sol-gel to substrate, aging and drying	3–55 MPa	Bone-like apatite formation in SBF, good biological activity. antifungal effects	Used for preparing thin films, possible for coating on the surface of particles of powder materials
Micro-Arc oxidation (Sedelnikova et al., 2017; Li et al., 2018)	Suitable for ceramic membrane, firm bonding, dense and uniform ceramic membrane	≈1–3 μm min^–1^ in thickness	5–44 MPa	Improving the adhesion of cells. Good antibacterial ability against *E. coli* and *Staphylococcus aureus*	Used for improving the surface roughness

### Chemical Vapor Deposition

Chemical vapor deposition represents a coating method to form a thin film layer on the substrate surface by chemical reaction of one or several vapor compounds or elements that contain the final film elements (Marsh et al., 2010). It has been used in the inorganic synthetic chemistry to prepare inorganic materials like carbon nanotubes, graphene, TiO_2_, etc. (Somani et al., 2006), the final product can be carefully controlled, both quantitatively and qualitatively. The facts have shown that the technology is very successful in industrial applications. However, their applications on titanium alloy substrate for biomedical surface modification is still limited.

Chemical vapor deposition methods are mainly used for complex workpieces and inner hole coating. Coatings prepared by the CVD method usually show high osteogenic activity, which has a certain potential for orthopedic applications. Giavaresi et al. (2003) used a metal-organic chemical vapor deposition (MOCVD) method to prepare the titanium oxide layer on pure titanium. Ti/MOCVD exhibited higher ALP activity than the control group, which means it has a higher potential for bone implantation. Subsequently, Du et al. (2016) successfully deposited Si-doped TiO_2_ nanowires on the TiSi_2_ layer by atmospheric pressure chemical vapor deposition (APCVD). It is not only showing higher hydrophilic activities but also has great importance in the field of doping. Related to the previous works, Xu et al. (2016) grafted a thin graphitic C_3_N_4_ (g-C_3_N_4_) layer on aligned TiO_2_ nanotube arrays (TiNT) by CVD. The binary nanocomposite coating shows excellent bactericidal efficiency. Glycidyl methacrylate (GMA) is a chemically versatile reagent through a ring-opening reaction (Mao and Gleason, 2004; Kang et al., 2014). Hence, in the research of Park et al. (2015), dot-patterned GMA-coated titanium implants notably displayed higher ALP activities, while displayed increased protein adsorption and higher calcium deposition. Furthermore, based on the previous study, Youn et al. (2019) added recombinant human bone morphogenic protein-2 (rhBMP2) as osteoinductive agents on GMA-coated titanium. From *in vitro* analysis, they found its good osteogenic activity without any cytotoxicity. Scarce information exists on the effect of amino group plasma-enhanced CVD on nerve regeneration. Hence, Zhao et al. (2018) introduced the amino group onto the titanium disk. Although, it exhibited the best cell attachment performance, it inhibited the expression of the key growth factors like glial cell-derived neurotrophic factor (GDNF) and neurotrophin nerve growth factor (NGF) *in vitro*, at least within a week. Tantalum coating on porous Ti-6Al-4V scaffold was investigated by Li et al. (2013), they found the better bone ingrowth within the coated scaffolds, indicating the potential for orthopedics. Interestingly, Ji et al. (2016) compared the adhesion of *Streptococcus mutans* on polished titanium (control group), magnetron sputtering titanium, and plasma nitriding modified titanium samples. There is not any clear difference between the treated samples and the control group. Gu et al. (2018) indicated the influence of heat treatment on the bonding strength and osteoinductive activity of monolayer graphene sheets with the antibacterial and osteoinductive properties.

In summary, the utilization of the CVD methods is not as common as the physical methods mentioned earlier. It may be because of its high reaction temperature that leads to a low deposition rate, also in this method the gas source and exhaust gas have certain toxicity, which may be harmful to the subsequent implantation process. Despite this, the coatings made by CVD usually have good quality, and their purity and density can be controlled. It has been used in industries like electronics, automobiles, aviation, and aerospace. However, vapor deposition equipment is more expensive, and some processes have higher film forming temperatures, which may adversely affect the structure of the substrate. In addition, some process line-of-sight film forming methods are more difficult to form on small shaped parts and need to be improved. In the future, the preparation of copolymer and inorganic coatings by CVD should be studied in depth to form a bacteriostatic surface.

### Sol-Gel

**FIGURE 8:**
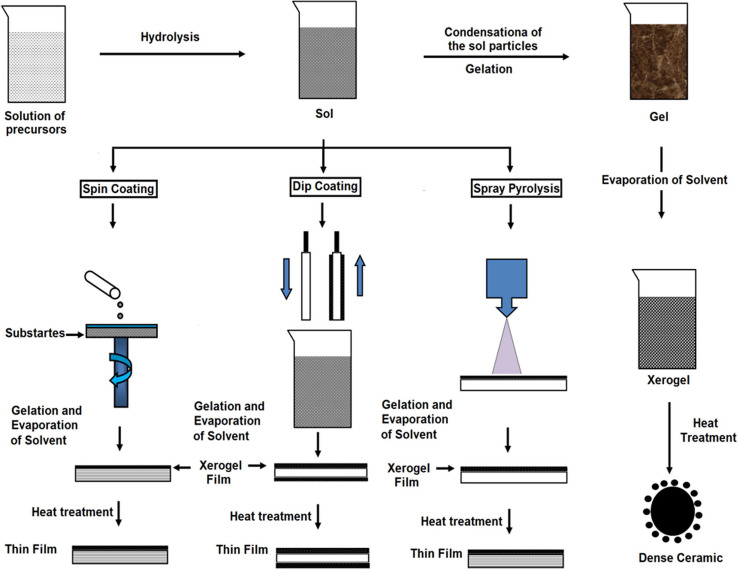
Schematic representation of sol-gel. Reproduced from Kaliyannan et al. (2020) with permission.

The Sol-gel technique is widely implemented to produce multifarious oxide films. This kind of method has the following advantages: simple fabrication environment, reliability of the consuming equipment, high uniformity of films, and utilization of different sizes of the substrate (Hench and West, 1990). The main factor that affects the sol-gel method is pH, chemical equilibrium, substrate-precursor interface, time, etc. (Wang and Bierwagen, 2009). Figure 8 is the schematic representation of sol-gel.

Ti, the dominant material for orthopedic application now, maybe impairing physical integrity like change its hardness and flexural modulus after sol-gel treatment. In order to solve this problem, Greer et al. (2016) evaluated coatings properties at different annealing temperatures and concluded that although the ductility decreased, 500°C was the optimal annealing temperature. TiO_2_ coatings have good physical properties as follows: high surface hardness, good wear resistance, low friction coefficient, and excellent corrosion resistance. Çomaklı et al. (2018) compared TiO_2_ films produced by sol-gel and successive ionic layer adsorption and reaction (SILAR) methods, the former exhibited better wear and corrosion resistance than the latter. Titania containing silver was deposited on TiSi alloys and commercially pure titanium (CP-Ti) by Horkavcova et al. (2017) and Yetim (2017), respectively. The results showed no cytotoxicity and excellent corrosion resistance, which means these materials are potential candidates for orthopedic application. Moreover, Ziabka et al. (2020) confirmed that this coating can be used in the veterinary treatment of bone fractures. In addition, doping with silver, TiO_2_ often forms a double-layer coating with HA. As mentioned before, the use of HA promotes bone formation, and in addition, proper chemical homogeneity can be obtained through sol-gel technology (Domínguez-Trujillo et al., 2018). Mohammed Hussein and Talib Mohammed (2019) prepared bilayer TiO_2_/HA coating, which has good corrosion protection with advanced crystallization and nano-scale homogeneous surface morphology. To enhance the adhesion strength of HA coatings sintered at low temperatures, Robertson et al. (2019) formed titania nanotubes through anodization. Azari et al. (2019) did further research and produced a functionally graded HA-TiO_2_ on Ti-6Al-4V alloy substrate and improved the adhesion and cohesion of the single-layer coating. Meanwhile, other strategies have been adopted to deal with the shortcomings of HA. Zinc substituted hydroxyapatite/bismuth (Zn-HA/Bi-HA) biphasic coatings were fabricated through sol-gel and dip-coating processes by Bi et al. (2020), which exhibited the most positive effect on osteoblast proliferation.

**FIGURE 9:**
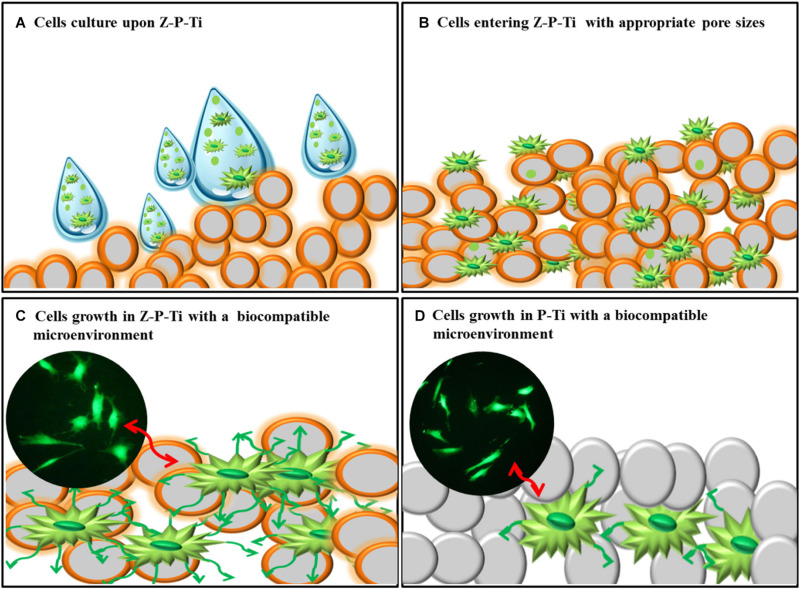
The mechanism of interaction between Z-P-Ti and the cell surface. Reproduced from Lee et al. (2017) with permission. **(A)** Cells culture upon Z-P-Ti. **(B)** Cells entering Z-P-Ti with appropriate pore sizes. **(C)** Cells growth in Z-P-Ti with biocompatible microenvironment. **(D)** Cells growth in P-Ti with biocompatible microenvironment.

Moreover, bio-inert ceramics like silicon dioxide and zirconia attracts a wide range of attention because of their stability in the human body. It also exhibits excellent load-bearing capacity and high cell viability. Lee et al. (2017) made zirconia-coated porous-Ti (Z-P-Ti) by the hydrothermal method, and then the sol-gel method was used. Among the samples, Z-P-Ti_55 (Ti samples with 55 wt.% additions of NaCl) exhibited excellent load-bearing capacity and high cell viability. Figure 9 is the mechanism of interaction between Z-P-Ti and the cell surface. Sandblasting Al_2_O_3_ combined with ZrO_2_ sol-gel layer was obtained by Lubas et al. (2018), providing a stable bond. Romero-Gavilan et al. (2018) prepared silica hybrid sol-gel coating (35M35G30T) on Ti, it can adsorb a large number of complement proteins. These proteins are involved in maintaining cell renewal, healing, proliferation and regeneration, and many other processes, which might be related to their intrinsic bioactivity. The addition of strontium (Sr) could affect their interactions with cells and proteins. Thus, Romero-Gavilan et al. (2019) applied a silica-hybrid sol-gel network doped with SrCl_2_ as a coating on Ti. In *in vitro* analysis, the coating containing Sr is more abundant in proteins involved in the coagulation process. Besides, the gene expression of ALP and TGFβ was enhanced in the MC3T3-E1 cells.

Recently, organic-inorganic composite coatings have also received a lot of attention, which is a suitable candidate for metallic prosthetic equipment. Catauro et al. (2018) synthesized coating from a multicomponent solution. Higher vitality of cells seeded on the coated samples was recorded and the higher HA nucleation was detected on the CP-Ti surface after soaking in SBF, which was also happened in the research of Aghajanian et al. (2019). They coated the porous titanium surface with forsterite/poly-3-hydroxybutyrate (P3HB) nano-biocomposite, this coating inhibited the excessive pH increment of the SBF. Moreover, Palla-Rubio et al. (2019) found that different amounts of chitosan and tetraethyl orthosilicate (TEOS) could modulate silicon release in hybrid silica-chitosan coatings, which plays a critical role in osteoregeneration. Based on the former research, Ballarre et al. (2020) added gentamicin to the chitosangelatin/silica, aimed at extending the bioactive effect. Based on, the sol consists of ZrO_2_, TiO_2_, Li^+^, and polyethylene glycol (PEG), Alcázar et al. (2019) evaluated the biocompatibility of hybrid coatings and found that the modified titanium surfaces have higher cellular growth. El hadad et al. (2020) have developed a new hybrid nanocomposite coating based on organofunctional alkoxysilanes precursors and phosphorus precursors, which prove that the presence of phosphorus at the molecular level can lead to the enhancement of biocompatibility. Simultaneously, Garcia-Casas et al. (2019) also deemed that an intermediate quantity of organophosphate showed the ability to enhance the mineralization of the substrate, which is why it was considered as the most suitable candidate for metallic prosthetic equipment. The drawbacks of pure HA were overcome by adding multi-minerals with the combination of PSSG polymer as hydroxyapatite/sorbitol sebacate glutamate (MHAP/PSSG) composite (Pan et al., 2019). Interestingly, Melatonin (MLT), used primarily to regulate the circadian rhythm and its role in bone regeneration and inflammation has been studied. Cerqueira et al. (2020) used sol-gel coatings as a release agent for MLT on a titanium substrate, they found that it didn’t improve the ALP activity, but has the potential in the activation and development of pathways. Based on the sol-gel coating, Toirac et al. (2020) added two different fungicides (fluconazole and anidulafungin) directly both of them exhibited anti-fungal properties.

At present, more research is conducted on the composition control of the sol-gel method than the process parameter control. These sol-gel coatings greatly enhance the corrosion protection and the migration of the metal matrix, thereby reducing the incidence of prosthesis rejection. Like other thermal deposition methods, it needs to consider the impact of thermal effects, so its current clinical use is subject to certain restrictions. There are extensive studies on the preparation of titanium dioxide, bio-inert ceramics, and organic-inorganic composite layers. For the titanium dioxide coating, the performance can be improved by doping other elements or improving the structural design of the composite coating with HA. For organic-inorganic composite coatings, in the future, it is possible to assess the comprehensive biocompatibility experiments by increasing the types of coating raw materials and adjusting the ratio.

### Micro-Arc Oxidation

Micro-arc oxidation, is developed based on anodizing technology. The MAO process mainly relies on the matching adjustment of the electrolyte and the electrical parameters. The process is done under the instantaneous high temperature and high pressure generated by the arc discharge, on the surface of Al, Mg, Ti, and other valve metals and their alloys. A modified ceramic coating produced by MAO is mainly composed of base metal oxides and supplemented with electrolyte components (Han et al., 2003; Li et al., 2004). It has the advantages of simple process, small area, strong processing capacity, high production efficiency, suitable for large industrial production, environmental protection, etc. (Liu et al., 2015; Wang et al., 2015).

**FIGURE 10:**
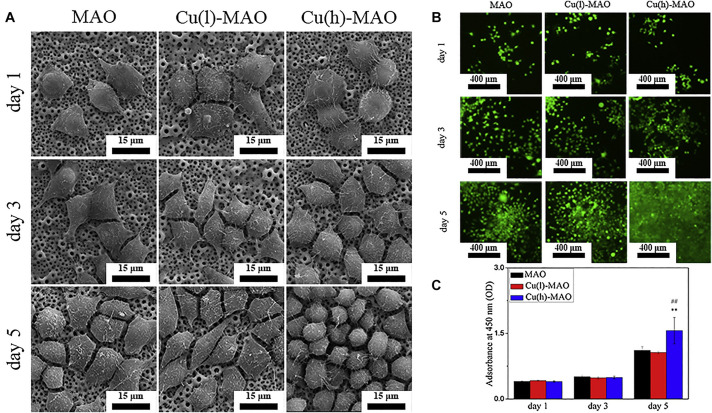
**(A)** The morphology of macrophages cultured on the surface of various materials for 1, 3, and 5 days. **(B)** Calcein-AM staining and **(C)** CCK-8 results show that Cu(h)-MAO surface promotes the proliferation of macrophages. Reproduced from Huang et al. (2018) with permission.

**FIGURE 11:**
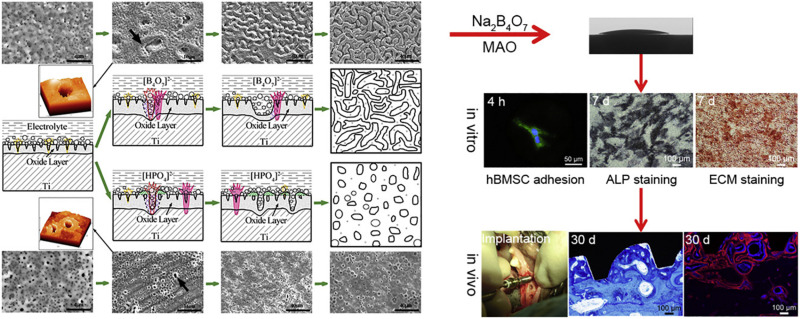
The forming mechanism and biological performance evaluation of the micro-arc oxidized TiO_2_ coatings with the “cortex-like” structure and the “volcanolike” structure. Reproduced from Li et al. (2018) with permission.

According to the principle of plasma-electrolytic oxidation, MAO can create a macro-porous and firmly adherent TiO_2_ film on the Ti substrate, which got a lot of attention. Some organic substances coated on the layer can make a balance between antibacterial and cell compatibility (He et al., 2018). Additionally, bioactive elements, like B, Ag, Ca, and Sr can be incorporated with TiO_2_ coating to enhance its bioactivity and biological properties. Huang et al. (2016, 2018) prepared boron-incorporated TiO_2_ coating (B-TiO_2_ coating) and Cu-containing TiO_2_ coatings successively. Specifically, the change of the chemical properties of the surface of B-TiO_2_ coating and release of B ions from its surface is believed to be the main reason for the improvement of ALP activity and cell differentiation. In the latter research, although the incorporation of Cu did not change the surface morphology and roughness, it still improved the macrophage-mediated osteogenesis and sterilization ability (Figure 10). Zhang et al. (2020) also fabricated Cu-TiO_2_ through a one-step MAO in a solution containing ethylene diamine tetraacetic acid cupric disodium (Na_2_CuEDTA) that has a two-layered coating consisting of TiO_2_ and porous Ca, P-rich outer layer containing nano-sized HA crystals. Subsequently, they investigated the enhanced antibacterial property and osteogenic activity of Zn-TiO_2_ coating fabricated by one step MAO method (Zhang et al., 2019). This structure improved the proliferation and differentiation of osteoblasts and slightly enhanced the antibacterial capability relative to its relatively higher Cu content. Ag-incorporated TiO_2_ coatings were prepared by Lv et al. (2019), the resultant film exhibits significantly improved antibacterial capability and bone-forming capacity with the increase of Ag_2_O nanoparticles in the electrolyte, also it has a slightly upgraded cytotoxicity behavior relative to polished Ti substrate. Li et al. (2020) incorporated Ca and Sr, which are good for bone reconstruction, into the MAO coating. This coating has a highly porous and super-hydrophilic layered structure, which showed excellent promoting effects in the proliferation of human bone marrow-derived mesenchymal stem cells (hBMSC). It is also a good way to combine MAO with other processes to improve the performance of the coating. Therefore, Tang et al. (2020) prepared BaTiO_3_ on the surface of TiO_2_ produced by MAO through a hydrothermal reaction. In the early period after bone implantation, the piezoelectric effect of this coating may play a positive role in bone growth and bone integration. High energy shot peening (HESP) pretreatment can be used to enhance the stability and bioactivity of the TiO_2_ coatings fabricated by MAO, Shen et al. (2020) used this method to increase the effective doping of Ca & P elements on the surfaces. Novel “cortex-like” coatings were investigated by Li et al. (2017, 2018), they have studied a macro/micro/nano triple hierarchical structure and micro/nano dual-scale structured TiO_2_ coating on Ti. Results proved that the “cortex-like” structure significantly promotes the cell adhesion, diffusion, and differentiation and increases the matrix mineralization. The graphic abstract and schematic diagram of the “cortex-like” TiO_2_ were shown in Figure 11.

The incorporation of Ca and P species into the TiO_2_ surfaces can cause the biocompatible compound formation. Thus, plenty of studies focused on obtaining HA-containing coating on titanium and its alloys. Karbowniczek et al. (2017) proved that in the electrolyte containing disodium hydrogen phosphate and calcium acetate hydrate, with the Ca/P ratio of 2/1 the coated Ti6Al7Nb alloy achieved the best combination of bioactivity and mechanical properties. Through a two-step method, it is also possible to produce an oxide layer with micro-pores and bio-active elements by MAO on a surface with macro-porosity (Costa et al., 2020). Similarly, Durdu et al. (2018) combined thermal evaporation-physical vapor deposition (TE-PVD) and MAO. In addition to higher hydrophilicity, the uniform and dense apatite distribution were observed on the Ag-incorporated coatings. Sedelnikova et al. (2017) deposited wollastonite-calcium phosphate (WeCaP) on the pure titanium, revealing the identical dependencies of coating thickness variation, surface roughness, and adhesion strength with process voltage. Interestingly, calcium-rich waste eggshell was used to produce HA coating on Ti-6Al-4V, which is in good agreement with that of bone.

As a hot spot technology for surface modification, MAO was used in lots of research schemes including the preparation of titanium dioxide and HA layers. The enhanced surface hydrophilicity of the porous coating prepared by the MAO method can stimulate the interaction between the implant and the surrounding biological environment, and it also brings excellent antibacterial properties due to the presence of metal ions. Although the anodic oxidation technology is convenient and economical, its bonding strength with the titanium matrix needs to be further improved. In future research, in addition to combining with other preparation methods, the structural design of the coating should be developed, such as the multi-level structure designs, multi-scale coating, or coating with novel surface morphology.

## Conclusion

Titanium and its alloys are the most commonly used materials for permanent implants, especially in application with direct contact with the bone, teeth, and bodily fluid. Numerous techniques exist to modify titanium and its alloys surfaces, their different mechanisms, procedure, and targets were listed in this review and with the goal for further clarification of how to choose the corresponding surface modification process and to select its optimum parameters for different demands.

This article reviews the main physical and chemical surface modification techniques for Ti related biomaterials, such as plasma spray, PIII, PIII&D, PVD, CVD, sol-gel, and MAO. Although these methods have been applied in practice and achieved some results, they still have some deficiencies, like the bonding strength still needs to be improved, the influence of thermal effects is eliminated, and how to compromise between toxicity and biological performance, etc. Future studies must be focused on designing the basic new methods or the combination of a variety of surface modification methods to play a synergistic effect and combine their advantages to conquer the deficiencies. On the other hand, the structure and composition of the composite coating can be tailored in order to achieve excellent biomedical performance.

## Author Contributions

TX and SA wrote the main part of the manuscript. SL, JL, and YT greatly contributed to the physical methods parts. SA made major contributions particularly in planning the tables. TX, SA, and XS made significant contribution to the revision stage. TX, XS, LL, and BZ prepared and formulated the references. All the authors contributed to the article and approved the submitted version.

## Conflict of Interest

LL and BZ were employed by the company Chengsteel Group Co., Ltd., HBIS Group Co., Ltd. The remaining authors declare that the research was conducted in the absence of any commercial or financial relationships that could be construed as a potential conflict of interest.
